# Epstein Barr virus: a prime candidate of breast cancer aetiology in Sudanese patients

**DOI:** 10.1186/1750-9378-9-9

**Published:** 2014-03-07

**Authors:** Zeinab A Yahia, Ameera AM Adam, Magdeldin Elgizouli, Ayman Hussein, Mai A Masri, Mayada Kamal, Hiba S Mohamed, Kamal Alzaki, Ahmed M Elhassan, Kamal Hamad, Muntaser E Ibrahim

**Affiliations:** 1 Institute of Endemic Diseases (IEND) Unit of Disease and Diversity, University of Khartoum Medical Campus, P.O. Box 102, Khartoum, Sudan; 2 Faculty of Medicine, University of Khartoum, Khartoum, Sudan

**Keywords:** Sudan, Epstein Barr virus (EBV), Breast cancer, DNA methylation

## Abstract

Breast cancer is the commonest cancer in Sudanese women. Reported genetic alterations in the form of mutations in tumor suppressors are low in frequencies and could not explain the peculiarities of the diseases including its focal nature. Potential contributors disease aetiology include oncogenic viruses such as Epstein-Barr virus (EBV), an established culprit of nasopharyngeal carcinoma, one of the most frequent cancers in Sudan.

In this study, DNA was extracted from malignant tissue samples and healthy tumour-free tissue from the same breast. Polymerase chain Reaction (PCR) was used to amplify two genes encoding for EBV viral proteins. The presence of Epstein-Barr virus and its cellular localization was confirmed by in situ hybridization (ISH) for Epstein-Barr encoded small RNAs (EBERs). Given the reported low frequency of mutations in *BRCA1* and *BRCA2* in Sudanese breast cancer patients, the methylation status of six tumor suppressor genes was investigated using methylation specific PCR. EBV genome was detected in 55.5% (n = 90) of breast cancer tissues as compared to 23% in control tissue samples (*p =* 0.0001)*.* Using ISH, EBV signal was detected in all 18 breast cancer biopsies examined while all five normal breast tissue biopsies tested were negative for EBV. Of six tumour suppressor genes investigated *BRCA1*, *BRCA2*, and *p14* appeared to be under strong epigenetic silencing.

In conclusion, we present evidence of a strong association between EBV and breast carcinoma in Sudanese patients, and considerable epigenetic silencing of tumor suppressors that may likely be an outcome or an association with viral oncogenesis.

## Background

Breast cancer is the most frequent malignancy among women in Sudan and worldwide. Despite the public health significance of the condition there are few well defined risk factors associated with the disease which could help explain its high incidence.

Infections with oncogenic viruses have been investigated as possible risk factors for breast cancer aetiology including mouse mammary tumor virus (MMTV), Epstein-Barr virus (EBV) and human papilloma virus (HPV) [1]. EBV was the first human virus to be directly implicated in carcinogenesis. It is a common infection affecting over 90% of the world’s population [2]. EBV has been implicated in the pathogenesis of Burkitt’s lymphoma, Hodgkin’s disease, non-Hodgkin’s lymphoma, nasopharyngeal carcinoma, as well as leiomyosarcomas arising in immunocompromised individuals [3]. The exact mechanism by which EBV transforms cells is not fully understood, although it has been suggested that cell cycle proteins could be the target of such transformation mechanisms [4], akin to other oncogenic viruses. HPV proteins for instance interact with the cell cycle proteins p53 [5] and retinoblastoma [6].

In Sudan, breast cancer is characterised by a geographically focal nature, early onset and aggressive course of the disease [7]. *BRCA1*, *BRCA2* and *p53* mutations are infrequent in Sudanese breast cancer patients. Epigenetic changes are suggested as alternative mechanisms to account for the minor contribution of genetic alterations in three tumour suppressor genes, *BRCA1*, *BRCA2*, and *p53*, in both sporadic and familial breast cancer cases in Sudan [8].

Viruses are likely to play a role in inducing the two categorized forms of aberrant methylation, hypomethylation, and hypermethylation [9-11] but the exact mechanism involved is yet to be understood. With or without viral involvement, the picture seems to be complex enough; the methylation profiles of tumour suppressor genes appear to vary according to tumour type, and each tumour apparently displays a distinct ‘DNA hypermethylome’ [12].

The overall prevalence and endemicity of nasopharyngeal carcinoma, another EBV-associated cancer [13-15] and its high frequency among breast cancer families (Hamad, personal communication) prompted the current investigation of a possible association between EBV infection and breast cancer in Sudan.

## Methods

### Patients and sampling

92 biopsy specimens of breast carcinoma and 50 matched normal tissues adjacent to breast tumours were collected from operated individuals of varying ages from different hospitals in Khartoum State, Sudan, who had not yet received anti-cancer medications. Samples were collected after signing informed consent forms. The study was approved by the Ethical Review Committee of the Institute of Endemic Diseases, University of Khartoum.

All cancer and control tissues were divided into two parts, one was collected in 10% buffered formalin for histological examination and in situ hybridization (ISH), and the second was preserved as fresh tissue at -40°C for subsequent DNA extraction.

### DNA extraction and PCR for EBV

DNA was extracted using the chloroform method. DNA quality was assessed using 1% agarose gel electrophoresis and further evaluated in terms of the A260/280 ratio in a Nanodrop 1000 apparatus. EBV genome was detected by PCR using two primers that targets EBV nuclear antigen-4 (EBNA-4) 5′-GAGGAGGAAGACAAGAGTGG and 5′ GATTCAGGCGTGGTCCTTGG 3′ and latent membrane protein-1 (LMP-1) 5′CCGAAGAGGTTGAAAACAAA3′ and 5′GTGGGGGTCGTCATCATCTC 3′.

### In Situ Hybridization for Epstein-Barr encoded small RNAs (EBERs)

ISH was performed on 5 μm-thick paraffin embedded tissue sections. EBV peptide nucleic acid (PNA) Probe/Fluorescein (Dako) was used, and the signal was detected using the PNA ISH detection Kit. Hybridization lasted 1 hour at 55° Celsius (C) and was visualized by alkaline phosphatase (AP)-conjugated antifluorescein antibodies. Nitro blue tetrazolium (NBT)/Bromo chloroIndoyl phosphate (BCIP) (Dako, Denmark) was used as a substrate for AP. To test the sensibility of ISH, we used a negative and a positive control provided by the manufacturer, and an undifferentiated nasopharyngeal carcinoma specimen as a second positive control. A case was considered positive if the nucleus of a tumour cell stained dark blue or black.

### Methylation based PCR

Two types of PCR that detect methylation status were employed: a bisulphite conversion based test, followed by methylation-specific PCR, and an restriction digestion enzymatic methylation-sensitive PCR test. Bisulphite conversion was performed using MethylEasy™ Xceed (Human Genetic Signatures, North Ryde, NSW Australia) according to the manufacturer’s protocol. Enzymatic methylation-specific PCR was carried out to confirm the results in a subset of the samples and check the robustness of the analysis.

## Results

### Patients

Ninety two patients were enrolled in this study ranging in age between 25 and 84 years with a median of 54 years. Histologically, 25 (27%) patients suffered invasive lobular carcinoma, 47 (51%) infiltrative ductal carcinoma and 20 (22%) had carcinoma in situ.

### EBV detection

EBV genome was detected in 49 (53.3%) and 10 (11%) patients by LMP-1 and EBNA-4 PCR respectively. In the control tissues, EBV was detected in 12 (24%) patients using primers for LMP-1 while all control samples were negative when EBNA-4 primers were used (Table 1). Statistical analysis revealed a significant difference between cancer tissues and controls by both LPM-1 and EBNA-4 primers (*p* = 0.0001).

**Table 1:** **Detection of Epstein- Barr virus in breast cancer tissues and controls using EBNA-1 and LMP-1 primers and the *P *** **value for the significance of the difference**

**Primer**	**Breast cancer tissue**	**Controls**	*P*-Value*
**LMP-1**			
	49	12	0.001
Positive	43	38	
Negative			
**EBNA-4**			0.014
Positive	10	0	
Negative	82	50	

*Indicates Fisher Exact Test.

**Figure 1:**
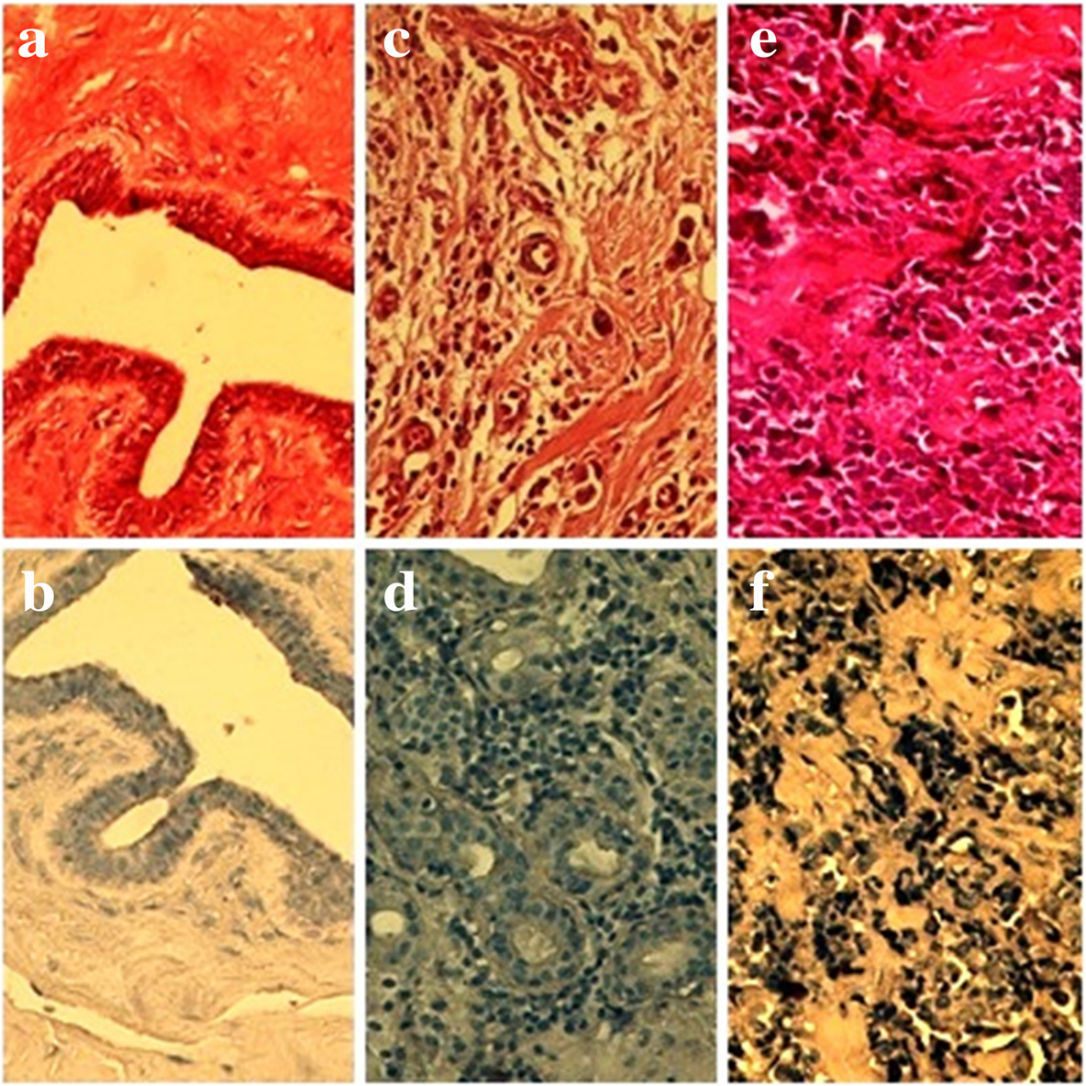
**Nuclear staining for EBER by in situ hybridization in infiltrative ductal carcisnoma of the breast and control tissues. a**. Normal duct staining for H&E. **b**. Normal duct negative for Epstein –Barr Virus by ISH for EBER. **c**. Infiltrative ductal carcinoma with individual cells in the stroma. **d**. Infiltrative ductal carcinoma of the breast, malignant cells showing positive Epstein-Barr virus by EBER. Positive signals are seen in the nuclei of tumor cells, but not in adjacent lymphocytes and normal cell. **e**. Invasive lobular carcinoma. **f**. EBV positive signal in tumour cells nucloi.

ISH was employed to confirm the presence of the EBV genome in malignant breast tissue. Using this technique, EBV was detected in all examined samples (18 biopsies) and its presence was confined to the malignant cells. In contrast, all five histologically normal tissues examined showed no signal for EBV (Figure 1).

### Methylation of tumour suppressor genes

The two methylation assays gave identical results in the subset of samples investigated, 33 tumour tissues and 4 control tissues. The methylation frequencies of the different genes were as follows: *BRCA1* 84%, *BRCA2* 84%, *p14* 81%, *p16* 15%, *hMLH* 18% and *MGMT* 12%. DNA extracted from whole blood samples was non-methylated in all genes. The methylation status differed between tumour types, in conformity to the literature. *BRCA1* and *BRCA2* were predominantly methylated in breast cancer while *p14* was more frequently methylated in oesophageal and colon cancer samples, ranging in frequency between 33.3% and 70% (Table 2).

**Table 2:** The methylation status of 6 genes namely BRCA1, BRCA2, p14, P16, hMLH, MGM2, in a sample of 23 breast cancer tissues, 7 colorectal, 3 esophageal and 4 control tissues (Blood)

**Gene**	**BRCA1**	**BRCA2**	**P14**	**P16**	**hMLH**	**MGM2**
**Cancer**		**Number hypermethylated**				
Breast (n = 23)	23	23	22	1	1	1
Colon (n = 7)	4	4	5	3	4	3
Oesophageal (n = 3)	1	1	ND	1	1	0
	84%	84%	84%	84%	84%	12%
Control (n = 4)	0	0	0	0	0	0

## Discussion

Infection with EBV virus is a frequent event. However, the reason why it is able to exert an oncogenic effect on some individuals while sparing others remains poorly understood. Factors involved in susceptibility to EBV-driven oncogenesis include host cell cycle proteins like p53 and others [16-19]. In this study, the correlation between breast cancer and the presence of the EBV genome was investigated. A highly significant positive correlation was found based on two PCR targets. Several studies have investigated this correlation, but reports are conflicting. The majority of studies document a wide range of frequencies for the presence of EBV in breast carcinoma [20]. Among 15 studies using PCR to detect EBV in breast tumours that we have reviewed, the virus was identified in a range of 0 to 66% of specimens. Prevalence was highest when PCR targeted the EBER and the reiterated BamH1W sequence [20-26], moderate when the target was LMP1 or EBNA4 [24] and lowest in investigations of EBNA1 [27,28]. These discrepancies indicate both the importance of the PCR target on the extent of the association and the natural causes and differences in aetiology between populations. Another source of variation is the segment of the EBV genome expressed. Xue *et al*. amplified EBV DNA in breast cancer tissues and used reverse transcriptase (RT) PCR to confirm expression of BART (BamH1A rightward transcription), LF3, EBNA1, BARF1, and BZLF1, establishing variation in the ability to detect molecular signals of presence/expression of these markers in the tumours [29]. Joshi *et al*. found that about 55% of breast cancer cases showed EBNA-1 expression in tumour cells, while all the controls with benign breast disease demonstrated no expression [30]. Our results are in agreement with studies that detected the virus using LMP-1 and EBNA-4 as targets for amplification.

Discrepancies in detection efficiency may also be due to technical differences or variation s between breast cancer subgroups. Generally, PCR is of value in detecting the presence of the virus in a particular neoplasm, but cannot directly demonstrate the association between the virus and the particular type of cancer. EBV DNA may be derived from tumour cells, surrounding stroma, or infiltrating lymphocytes. To address such concerns we compared tumour tissue with normal surrounding tissue from the same individual and furthermore used ISH to investigate cellular localization of the virus. Using ISH, we detected the EBV genome in all 18 breast cancer specimens investigated but could not detect the virus in healthy breast tissue, a much higher frequency compared with reports employing ISH. The confinement of viral DNA to tumour cells as we demonstrate here strongly suggests a viral contribution to breast cancer aetiology.

A recent transcriptome analysis of EBV host-viral regulatory interaction reported both oncogenes and tumor suppressors like CFOS and BRCA1 to be co-expressed during lytic phases of the viral replication indicating the role of EBV viral genome in cellular transformation [31]. The high frequency of EBV in breast cancer tissue, as reported here, might explain the low frequency of mutations in *BRCA1*, *BRCA*2 exon 11 and *p53* among breast cancer patients in Sudan [8]. These findings raise the stakes of the virus as an environmental and epigenetic culprit in breast cancer aetiology in Sudanese patients.

In line with these preliminary findings, the methylation status of 5 tumour suppressor genes (*BRCA1*, *BRCA2*, *p14*, *p16*, *hMLH* and *MGM2*) was investigated. The high frequency of epigenetic silencing in *BRCA1*, *BRCA2*, and *p14* suggests a potential influence of the virus on the methylation machinery, an oncogenic mechanism reported in other cancers but not yet in breast cancer [32,33]. The methylation of *p14* suggests a possible upstream mechanism that liberates the tumour genome from the control of *p53* and other major tumour suppressors thus annulling or minimizing the role of gene mutations. This mechanism was originally proposed by Esteller *et al*. in colorectal cancer on the basis of over-representation of *p14ARF* hypermethylation in tumours with wild-type *p53* compared to tumours harbouring *p53* mutations [34]. The existence of methylation signatures that dictate the transcriptional status of tumour suppressors or oncogenes as a prerequisite for cancer initiation or development is now commonplace and is believed to shape the biology of various tumours.

However, the inactivation of *p14* does not seem to be enough to replenish the loss of function of *p53* in cancer pathogenesis given the central network characteristics of the protein. We recently reported a striking risk association between the *p53* arginine allele and breast cancer in Sudanese individuals [35], an unusual example of a common polymorphism with a major effect (OR= 13). This polymorphism has a known geographic pattern of a north South cline [36], which brings into consideration the geographical conditioning of the association between EBV and nasopharyngeal carcinoma endemicity, a known EBV associated tumour, around the tropics [37].

In conclusion, we present unequivocal evidence supporting a major etiologic role for EBV in breast cancer pathogenesis in Sudanese patients. Our findings prove the presence of EBV in a high proportion of breast cancer samples in Sudanese patients. The localisation of the virus in malignant cells makes it a likely risk factor and a possible aetiological agent in breast carcinogenesis. If such a causal association of EBV with breast cancer is further established in functional terms, therapeutic implications might follow in light of the recent advances in EBV vaccination.

## Competing interests

Authors declare no competing interests.

## Authors’ contributions

ZAY, performed the laboratory and data analysis and contributed to writing the manuscript; AM A performed the in situ hybridization; ME, AH and MK, carried out the methylation assays and designed the tests; MAM and HSM, supervised the laboratory experiments and sampling procedures, KA; recruited patients and operated on them, AME, performed the histopathology and supervised the in situ hybridization assays, and KH, conceived of the study contributed to recruitment of the patients and their management; ME I designed the study and wrote the manuscript. All authors read and approved the final manuscript.
